# Genome-wide characterization and expression analysis of *Erf* gene family in cotton

**DOI:** 10.1186/s12870-022-03521-z

**Published:** 2022-03-22

**Authors:** Muhammad Mubashar Zafar, Abdul Rehman, Abdul Razzaq, Aqsa Parvaiz, Ghulam Mustafa, Faiza Sharif, Huijuan Mo, Yuan Youlu, Amir Shakeel, Maozhi Ren

**Affiliations:** 1 Zhengzhou Research Base, State Key Laboratory of Cotton Biology, Zhengzhou University, Zhengzhou, China; 2 State Key Laboratory of Cotton Biology, Key Laboratory of Biological and Genetic Breeding of Cotton, The Ministry of Agriculture; Institute of Cotton Research, Chinese Academy of Agricultural Science, Anyang, 455000 Henan China; 3 The Institute of Molecular Biology and Biotechnology, The University of Lahore, Lahore, Pakistan; 4 Center of Agricultural Biochemistry and Biotechnology, University of Agriculture, Faisalabad, Pakistan; 5 University Institute of Physical Therapy, The University of Lahore, Lahore, Pakistan; 6 Department of Plant Breeding and Genetics, University of Agriculture Faisalabad, Faisalabad, Pakistan

**Keywords:** ERF transcription factors, Segmental duplication, Tandem duplication, Synteny analysis, Expression profiles

## Abstract

**Background:**

AP2/ERF transcription factors are important in a variety of biological activities, including plant growth, development, and responses to biotic and abiotic stressors. However, little study has been done on cotton’s AP2/ERF genes, although cotton is an essential fibre crop. We were able to examine the tissue and expression patterns of AP2/ERF genes in cotton on a genome-wide basis because of the recently published whole genome sequence of cotton. Genome-wide analysis of *ERF* gene family within two diploid species (*G. arboreum* & *G. raimondii*) and two tetraploid species (*G. barbadense*, *G. hirsutum*) was performed.

**Results:**

A total of 118, 120, 213, 220 genes containing the sequence of single AP2 domain were identified in *G. arboreum, G. raimondii, G. barbadense* and *G. hirsutum* respectively. The identified genes were unevenly distributed across 13/26 chromosomes of A and D genomes of cotton. Synteny and collinearity analysis revealed that segmental duplications may have played crucial roles in the expansion of the cotton *ERF* gene family, as well as tandem duplications played a minor role. *Cis*-acting elements of the promoter sites of *Ghi-ERFs* genes predict the involvement in multiple hormone responses and abiotic stresses. Transcriptome and qRT-PCR analysis revealed that *Ghi-ERF-2D.6, Ghi-ERF-12D.13, Ghi-ERF-6D.1, Ghi-ERF-7A.6 and Ghi-ERF-11D.5* are candidate genes against salinity tolerance in upland cotton.

**Conclusion:**

Overwhelmingly, the present study paves the way to better understand the evolution of cotton ERF genes and lays a foundation for future investigation of ERF genes in improving salinity stress tolerance in cotton.

**Supplementary Information:**

The online version contains supplementary material available at 10.1186/s12870-022-03521-z.

## Background

Under confrontational environmental conditions such as pathogen attack, submergence, flood, salinity, drought, and cold, a precise expression pattern is shown by genes according to their physiological and biological processes [1]. Most importantly, the expression of functional genes in the genome is maintained by transcription factors (TFs). During biotic/abiotic stresses and developmental processes, the expression of transcription factors of particular genes is enhanced or suppressed by other proteins [2]. Transcription factors for playing a vital role as chief regulators in various biological processes are the most important target for crop engineering [3]. The *AP2/ERF* family is a large group of plant-specific transcription factors. This gene family having *AP2/ERF*-type DNA-binding domain of about 60–70 amino acids was first discovered in *Arabidopsis* homeotic gene, *APETALA2 (AP2)* [4]. *AP2, ERF, RAV,* and *DREB* are four subfamilies of *AP2/ERF* gene family. *AP2* subfamily is different from the other 3 (*ERF, DREB,* and *RAV*) subfamilies as it has double *AP2/ERF* domains while all three have a single *AP2/ERF* domain whereas *RAV* is different from *ERF* and *DREB* subfamily by having an additional B3 DNA-binding domain. Over the last 20 years, ERF family genes caught attention as overexpression of *ERF* genes in different plants directed to abiotic stress tolerance and pathogen resistance in transgenic plants [5, 6].

The *ERF* subfamily members regulate the expression of PR (pathogenesis-related) genes through binding to GCC-boxes (AGCCGCC). Further, ERFs are intricated in signaling pathways including salicylic acid, jasmonic acid, and ethylene pathways which are important for stress response and plant development [7–9]. Due to their coordinating ability with multiple signaling and hormonal pathways, ERFs are considered excellent entities for engineering biotic/abiotic stress tolerance in plants [9]. Several studies have shown the involvement of ERF gene expression under different stress tolerance conditions and tissues in plants [10–12].

Cotton is the main agro-industrial crop and is the source of the most important natural fiber used in textile production [13]. This plays a vital role in a global economy and is grown in more than seventy countries. The genus *Gossypium* contains 46 diploid (2n = 2x = 26) and 5 well-established and 1 purported tetraploid (2n = 4x = 52) species. It has been proposed that all diploid cotton species may have evolved from a common ancestor that subsequently diversified to produce eight groups, including groups A–G and K3 [14]. However, biotic and abiotic stresses badly affect the production and growth of cotton. So, the struggles to explore the molecular mechanism of stress to support stress tolerance in plants is real and of fundamental importance for cotton production [15]. Considering the importance of *ERF* family genes in crop improvement, genome-wide investigation of *ERF* gene-family in cotton can help us to understand the molecular mechanisms of resistance to stress, and thus aid in the development of cotton varieties, using transgenic technology, with greater tolerance to many adverse environments. The release of different cotton whole-genome sequence data, including *Gossypium arboreum* L. [16], *Gossypium raimondii* [17], *Gossypium hirsutum* L. [18], and *Gossypium barbadense* L. [19] has made it possible to systematically identify and analyze the cotton *ERF* genes on a genome-scale level. Here, we performed a comprehensive analysis of cotton *ERF* genes, including their gene structure, motif compositions, chromosome distribution, duplication patterns, and expression profiles. This study will provide valuable clues for the functional characterization of the *ERF* gene family in cotton.

## Methods

### Database and sequence retrieval

Gene sequences of *Arabidopsis AtERF* were retrieved from the TAIR (*Arabidopsis thaliana* database) [20]. Cotton genome database Cotton FGD was used for retrieval of *G. barbadense* (NAU), *G. hirsutum* (CRI), *G. raimondii* (JGI) and *G. arboreum* (CRI) genome sequences [21]. *ERF* domain sequence was used as a template to retrieve the probable domain homologs from the whole-genome sequence of *G. barbadense*, *G. hirsutum*, *G. raimondii,* and *G. arboreum* [22] through BLASTP at CottonGen (https://www.cottongen.org). All non-redundant hits with less than 1E-5 E-value were taken. Non-targeted and overlapping sequences were removed. Pfam30.0 (http://pfam.xfam.org/) database was used to retrieve hidden Markov Model (HMM) profile of the U-box domain (PF00847) [22], and retrieved results were used as a template to find out the candidate *ERFs* from the cotton genome protein database using HMMER3.0 [23]. Protein sequences, CDSs (coding domain sequences), and corresponding full-length sequences in the genome were obtained by using BLAST2.2.31+ (ftp://ftp.ncbi.nlm.nih.gov/blast/executables/blast+/LATEST/). Further, SMART (http://smart.embl-heidelberg.de/) and Pfam 30.0 [22] databases were used for additional analysis to ensure that each candidate protein contained a U-box domain and BUSCA was used for subcellular localization prediction [24, 25].

### Gene structure and conserved motif analyses

MEME (Multiple Em for Motif Elicitation) version 5.3.0 (http://meme-suite.org/tools/meme/), an online tool was used for the identification of conserved motifs of *ERF* proteins. TBtools software was used to predict gene structure and integrate phylogenetic trees and conserved motifs.

### Analysis of *Cis*-acting regulatory elements in promoter regions

A 1.5 kb of promoter sequence upstream from the transcription start site in each *Ghi-ERF gene* was extracted from the *G. hirsutum* genome database and analyzed using PlantCARE online software (http://bioinformatics.psb.ugent.be/webtools/plantcare/html/) to predict the putative cis-acting regulatory elements [26].

### Physical location of ERFs on the chromosome and phylogenetic tree

Mapcahrt was used to generate the distribution of cotton ERF on the chromosome. Genome annotation files were used to retrieve the GFF (general feature format) information of the cotton *ERFs*. The 671 protein sequences of all four species of cotton and 60 protein sequences of *Arabidopsis* were used for the phylogenetic tree. The Clustal Omega (https://www.ebi.ac.uk/Tools/msa/clustalo/ [27]; was used to align *ERF* protein sequence and MEGA v7.0 [28] program was used to construct a neighbor-joining phylogenetic tree with 1000 bootstrap replicates.

### Gene duplication and micro-synteny analysis in *G. arboreum*, *G. raimondii, G. barbedense* and *G. hirsutum* L

To investigate the collinearity and to analyze the syntenic relationship among the four cotton species, the complete genome sequences of these cotton species along with respective genome annotation files were subjected to MCScanX tool [29]. TBtools software was used to visualize the obtained results. Multiple collinear scanning toolkits (MCScanX) and Dual Synteny Plotter software (https://github.com/CJ-Chen/TBtools/) [30] were used for the analysis of gene duplication events [29] and the syntenic relationship between the *ERF* genes among cotton species, respectively. Some previous studies showed the identification of homologous gene pairs according to MSA (multiple sequence alignment). Advanced circos was used to visualize the collinearity of homologous genes based on the homology between each species and their positions on the genome [30].

### Transcriptome data analysis expression analysis

To identify the expression pattern of identified ERF genes in various tissues of all the four cotton species, the RNA-seq expression data of *G. arboreum*, *G. raimondii*, *G. barbadense*, and *G. hirsutum* were downloaded from NCBI. The raw expression reads data from various anatomical tissues and various time-points of ovule and fiber of *G. arboreum* cultivar Shixiya1 and *G. hirsutum* accession TM-1 were retrieved from NCBI-Bioproject PRJNA594268 [31]. Furthermore, RNA-seq expression data of *G. hirsutum* accession TM-1 and cultivar Hai7124 were retrieved from NCBI-Bioproject PRJNA490626 [32] for *G. barbadense*. Similarly, raw expression data of strain Ulbr of G. raimondii were obtained from NCBI-Bioproject PRJNA171262 [33]. The program hisat2 [34], was used for mapping reads, cufflinks (version: 2.2.1) [35], were used to analyze gene expression levels, and fragments per kilobase million values were used to normalize gene expression levels, then the results were log-transformed and a heatmap was generated by MeV [36].

### qRT-PCR

Cotton genotype (Eagle-2) was obtained from Four Brothers hybrid seed company Lahore, Pakistan and permission was granted. The cotton (Eagle-2) seeds were germinated on a wet filter paper for 3 days at 25 °C, and then transferred to a Hoagland nutrient solution (Supplementary file 1) [37] in a greenhouse with 60–70% humidity, 14 h photoperiod, and 28 °C day/night temperature. Over a period of 6–8 weeks, the plants were placed in a greenhouse under controlled conditions. The selected cultivar Eagle-2 was exposed to salt stress at 0 dS/m, 10 dS/m, and 15 dS/m at two leaves stage. Newly emerged leaves of the cultivar Eagle-2 were taken after a regular interval of 1 h, 3 h, 6 h, 12 h, and 24 h for the extraction of RNA (RNAprep Pure Plant Kit by Tiangen, Beijing, China) and freeze in − 80 °C. RNA concentration and integrity were observed on NanoDrop 2000 spectrophotometer (Thermo Scientific, USA) and 1% agarose gel electrophoresis and cDNA was prepared (PrimeScript® RT Reagent Kit, Perfect Real Time, Takara Biotechnology Co., Ltd., Dalian, China). Technically, three replicates of each sample were used. The mean expression of mRNA was measured using qRT-PCR (Maxima SYBR Green)/ROX qPCR Master mix (2X), cat#K0221, Thermo scientific, USA. Gene-specific primers were used for the amplification of all the genes and GAPDH was used as an internal control for gene normalization.

## Results

### Identification, sequence analysis and phylogenetic tree of *ERF* genes in *G. arboreum, G. raimondii, G. barbadense* and *G. hirsutum*

**Fig. 1:**
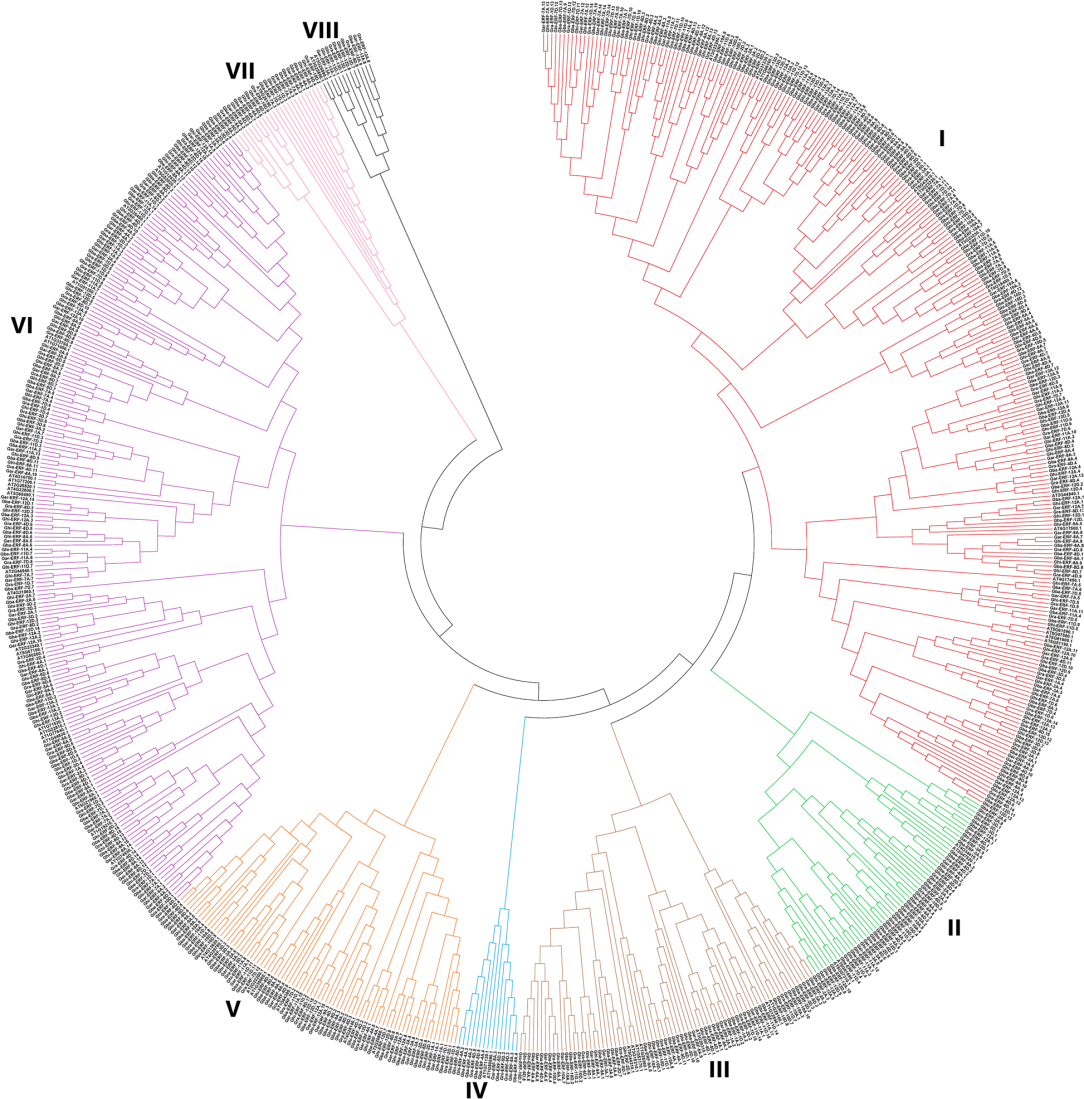
Phylogenetic tree of the *ERF* genes family of cotton and *Arabidopsis*. Bootstrapping values are indicated as percentages along the branches. The different background colors indicate different groups

A total of 118, 120, 213, 220 genes were identified in *G. arboreum, G. raimondii, G. barbadense,* and *G. hirsutum* respectively. To identify *ERF* genes, the sequence of *ERF* domain was a blast against the whole genome sequence of *G. arboreum, G. raimondii, G. barbadense,* and *G. hirsutum.* All non-redundant *ERF* genes were obtained from each species. The amino acid sequence of these 671 *ERF* genes was evaluated by Pfam software to confirm their reliability and the presence of *ERF* domains. Genes that lack *ERF* domain in the encoding protein sequences or truncated genes and the genes that were not annotated in their respective genome were deleted. The detailed information of selected genes for *G. arboreum*, *G. raimondii*, *G. hirsutum,* and *G. barbadense* are listed in (Supplementary file 1). According to locations on chromosomes the *ERF* genes in cotton were renamed, Gar-ERF-1A.1-13A in *G. arboreum,* Gar-ERF-1D.1-13D in *G. raimondii,* Gba-ERF-1A.1-13D in *G. barbadense,* Ghi-ERF-1A.1-13D in *G. hirsutum*. In *G. barbadense, 98 ERF genes were discovered on At sub-genome, and 115 genes were identified on Dt sub-genome. In G. hirsutum, 109 genes were identified in At sub-genome and 111 genes were identified in Dt sub-genome.* A phylogenetic (neighbour-joining) tree was constructed to determine the evolutionary relationship of ERF genes by using amino acid sequences of identified ERF proteins in *G. arboreum, G. raimondii, G. barbadense*, and *G. hirsutum* with corresponding 60 genes of *Arabidopsis* (Fig. 1). The evolutionary tree classified the *ERF* genes into 8 clades with well-supported bootstrap values. The clade one is the largest clade followed by clade VI and clade VIII has the lowest number of genes followed by clade IV. The results showed that ERF genes of four species of cotton and *Arabidopsis* were unevenly distributed in all clades. Only 2 genes from *G. arboreum,* and 2 from *G. raimondii,* were found in clade VIII.

### Chromosome distribution and gene duplication analysis

To discover the *ERF* genes distribution on chromosomes, each *ERF* gene was mapped on their corresponding chromosome according to gene information of the respective genome database. To further examine the evolution of *ERF* genes in four species of cotton, genome duplication events were investigated for WGD or segmental and tandem duplications.

**Fig. 2:**
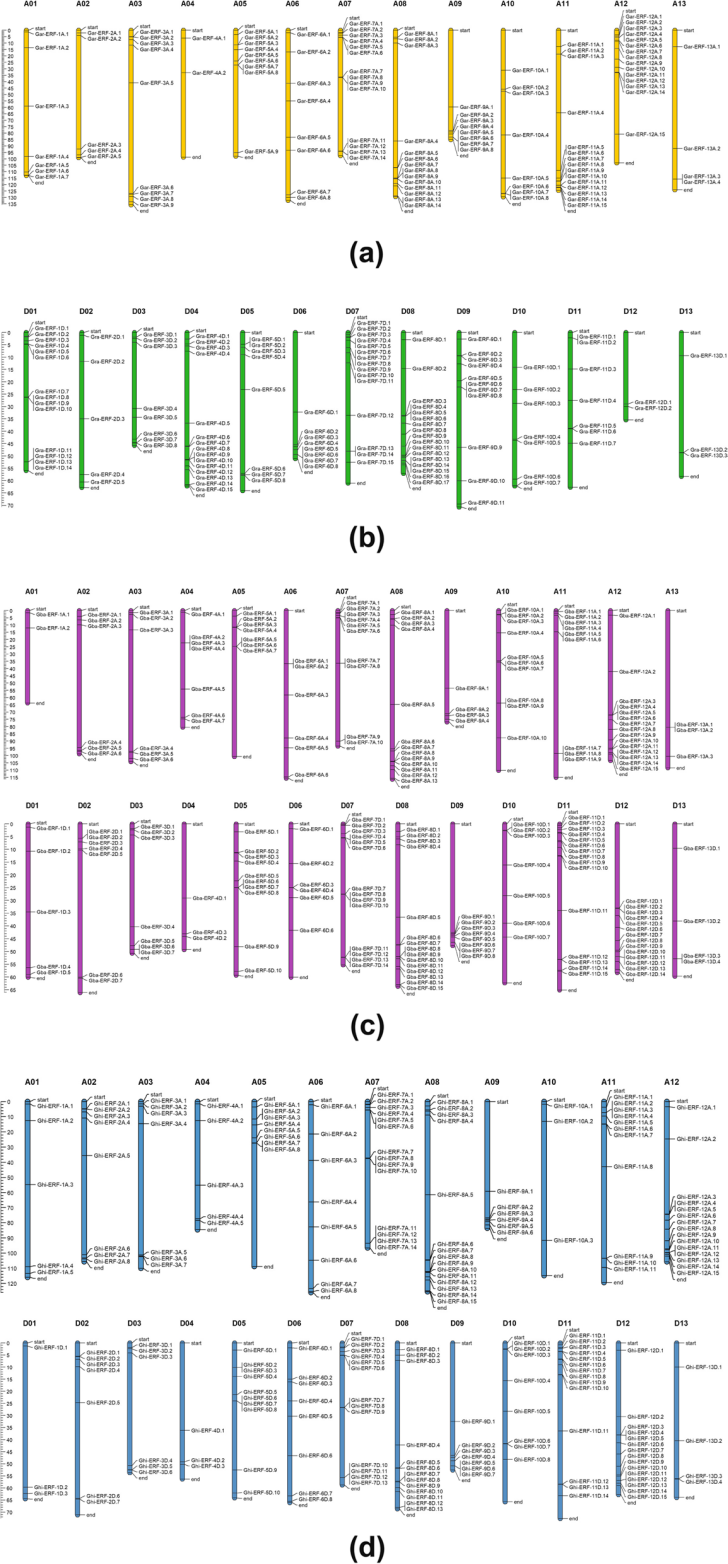
**a**, **b** Gene Location on chromosome of *G. arboreum* and *G. raimondii.* **c** Gene Location on chromosome of *G. barbadense*. **d** Gene Location on chromosome of *G. hirsutum*

**Fig. 3:**
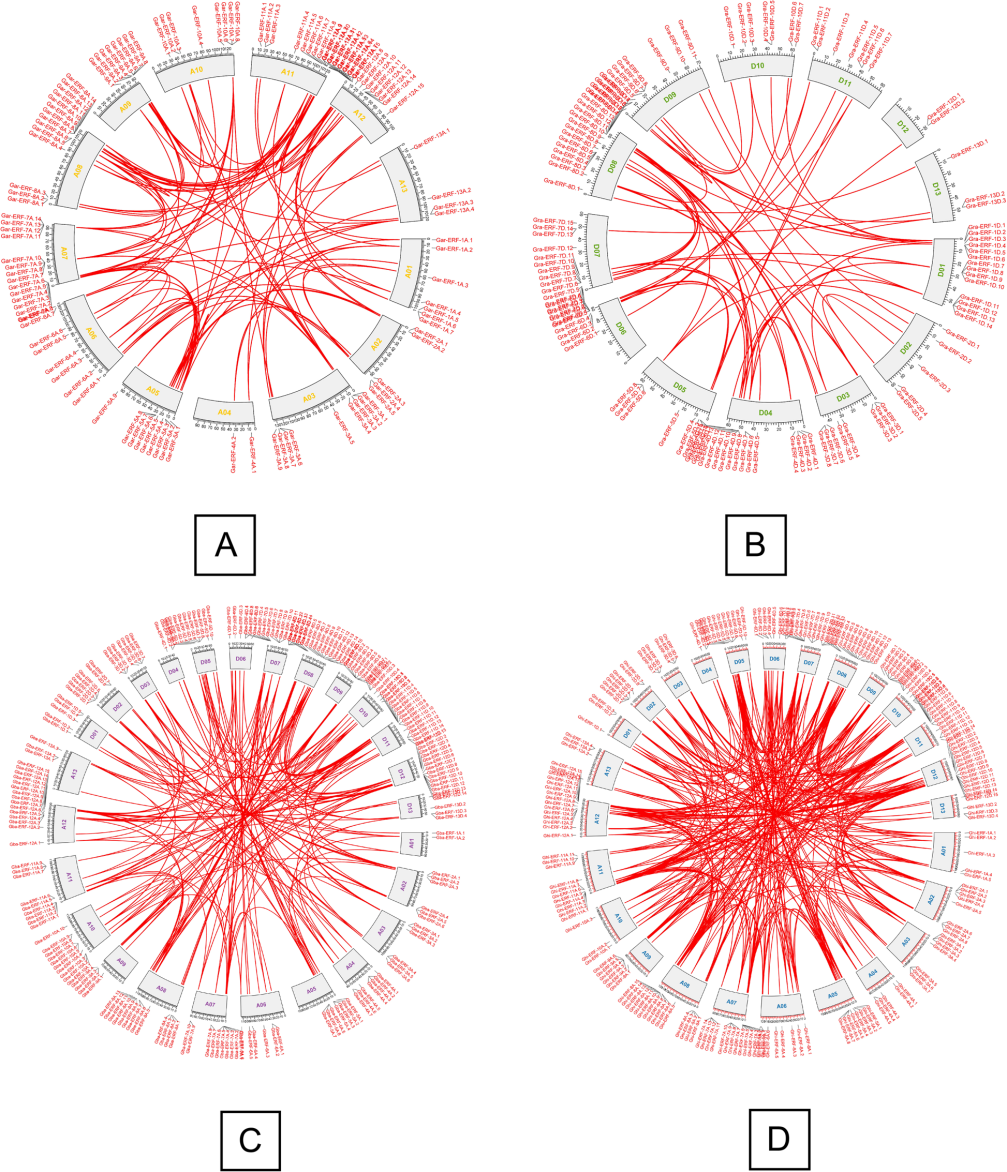
Duplicated ERF gene pairs identified in *G. arboreum, G. raimondii, G. barbadense* and *G. hirsutum*

In *G. arboreum,* 118 *ERF* genes were unevenly localized on all 13 chromosomes. The results showed that 7, 5, 9, 2, 9, 8, 14, 14, 8, 8, 15, 15, and 4 genes were located on chromosomes A1 to A13 (Fig. 2a). Chromosomes 11 and 12 both contained the highest number of *ERF* genes (15 *ERF*) whilst chromosomes 4 and 13 contained a lower number of *ERF* genes (2 and 4) respectively. To understand the expansion pattern of the *ERF* gene family in *G. arboreum,* a circos analysis was performed. The results revealed that 92 *ERF* genes have WGD or segmental duplication and are located on all chromosomes (Fig. 3A). The 13 ERF family genes were tandemly duplicated and distributed on chromosomes 2, 5, 7, 8, 9, and 12. Other 11 genes were dispersed in *G. arboreum* genome (Supplementary file 2)*.*

In *G. raimondii,* 120 ERF genes were distributed unequally on all 13 chromosomes (Fig. 2b). The number of ERF genes from chromosome D01 to D13 was 14, 5, 8, 15, 8, 8, 15, 17, 11, 7, 7, 2, and 3 respectively. The chromosomes D08 and D04 contain the highest number of genes 17 and 15 respectively. While chromosome D12 and D13 exhibited the lowest number of genes 2 and 3 respectively. Among120 ERF genes 74 genes have *WGD or* segmental duplications and were located unevenly on all chromosomes (Fig. 3B). The chromosomes D08 and D01 contain the highest *WGD or* segmentally duplicated genes 14 and 10 respectively. On chromosomes D12 and 13 only a single gene have segmental duplications (Fig. 3B). The genes *Gra-ERF-1D.6, Gra-ERF-1D.13, Gra-ERF-4D.3, Gra-ERF-4D.9, Gra-ERF-6D.6, Gra-ERF-6D.7, Gra-ERF-6D.8, Gra-ERF-7D.14, Gra-ERF-8D.15, Gra-ERF-9D.4, Gra-ERF-9D.7, Gra-ERF-9D.8*, and *Gra-ERF-13D.3* have tandem duplications (Supplementary file 2)*.*

In *G. barbadense*, 213 *ERF* genes were scattered on all 26 chromosomes of At and Dt sub-genomes. The *98 and 115 ERF genes were identified in At and Dt sub-genomes respectively* (Fig. 2C)*. In At genome of G. barbadense,* chromosome A12 had maximum (15) *ERF* genes whilst chromosome A01 and A13 have a minimum number of *ERF* genes 2 and 3 respectively. In Dt sub-genome, the chromosomes D08 and D11 contain the greatest number of (15) ERF genes and chromosome D04 had the lowest (3) *ERF* genes (Fig. 2C). The 182 *ERF* genes of *G. barbadense*, have segmental duplications and these were scattered unevenly on all 26 chromosomes. Out of these 182 genes, 42.75 and 57.14% belonged to At and Dt sub-genome respectively (Fig. 3C). Chromosome A13 has only single genes *Gba-ERF-13A.3* had segmental duplication. From At sub-genome, the genes *Gba-ERF-4A.3, Gba-ERF-4A.4, Gba-ERF-5A.6, Gba-ERF-6A.2, Gba-ERF-7A.4, Gba-ERF-7A.9, Gba-ERF-8A.4, Gba-ERF-10A.7, Gba-ERF-11A.8, Gba-ERF-11A.9, Gba-ERF-13A.1* and *Gba-ERF-13A.2* have tandem duplications *(*Fig. 3C *&* Supplementary file 2). Only three genes *Gba-ERF-7D.13, Gba-ERF-7D.14*, and *Gba-ERF-11D.13* from Dt sub-genome have tandem duplications (Supplementary file 2)*.*

*In G. hirsutum*, 220 *ERF* genes were unevenly distributed on all 26 chromosomes. Among these 220, 109, and 111 *ERF* genes were located in At and Dt sub-genome respectively (Fig. 2d). *In At genome of G. hirsutum*, the chromosomes A08 and A12 have maximum (15) *ERF* genes whilst the chromosomes A10 and A13 have the minimum number of *ERF* genes 3 and 4 respectively. In Dt sub-genome, chromosome D12 contains the greatest number of (15) *ERF* genes and chromosomes D01 and D04 have the lowest (3) *ERF* genes (Fig. 2d). The segmental duplications were found in 94.55% *ERF* genes of *G. hirsutum*, 49.52, and 50.48% genes were located in At and Dt sub-genomes respectively *(*Fig. 3D and Supplementary file 2)*.* Tandem duplications were found in 3.18% *ERF* genes and these genes were located on chromosomes A07, A08, D07, D08, D09, and D11.

**Fig. 4:**
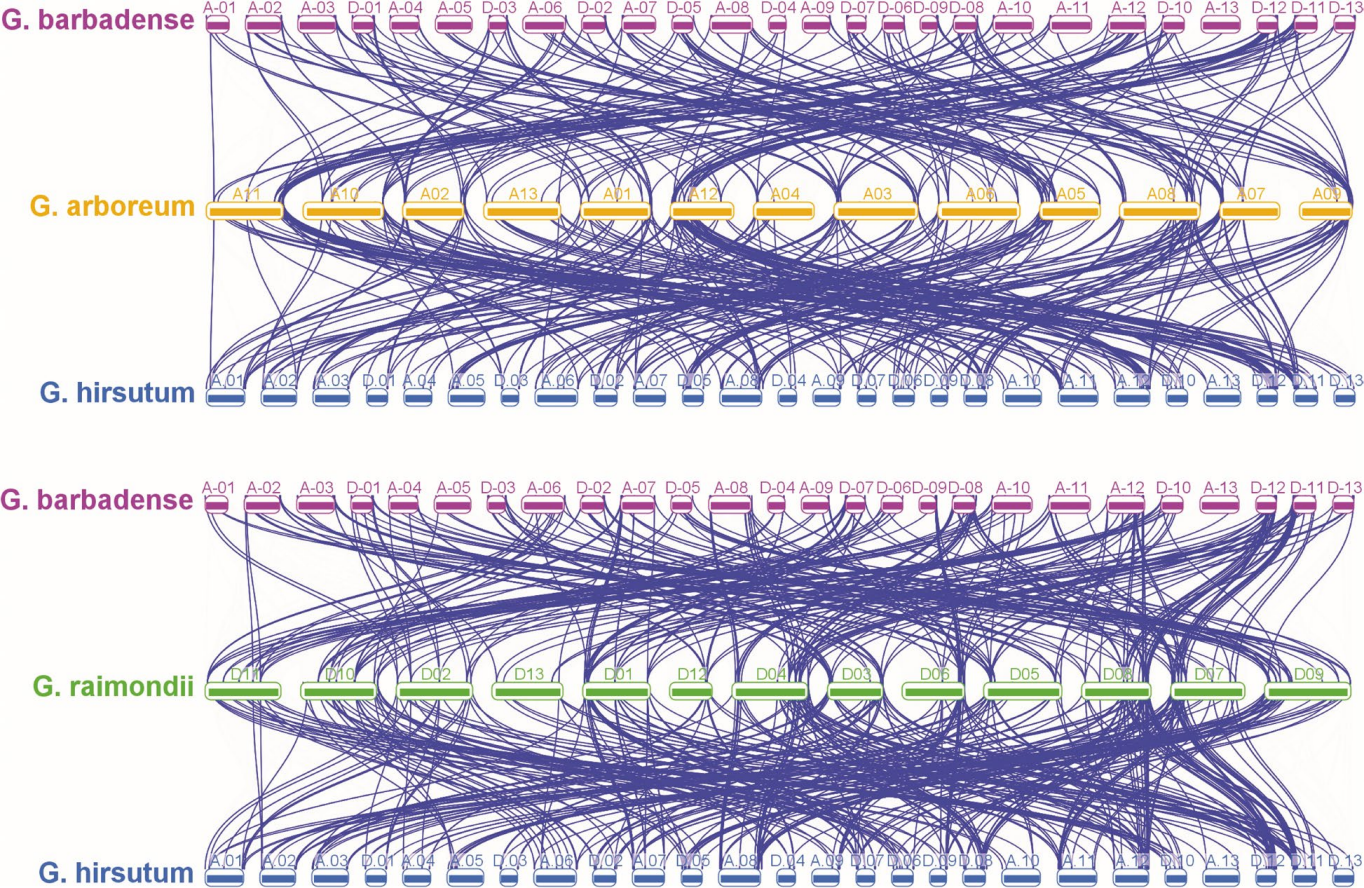
The sub-genome distribution and synteny analysis of cotton *ERF* genes. The blue lines indicate duplicated *ERF* pairs, the gray lines indicate collinear blocks

The *G. hirsutum* and *G. barbadense* are evolved due to hybridization between an A-genome species (*G. herbaceum* or *G. arboreum*) and a D-genome genome (*G. raimondii*) [38]. To understand the evolutionary relationships of *ERF* genes, a relative syntenic map of *ERF* genes from the four cotton species was fabricated (Fig. 4). Synteny analysis showed several gene loci that are highly conserved between the At and Dt sub-genomes of both tetraploid cotton species. According to our MCScan analysis, 237, 105 duplication gene pairs were found between diploid *G*. *arboreum* and tetraploid *G*. *barbadense, G*. *hirsutum* respectively. 393, 307 duplication gene pairs were found between diploid *G*. *raimondii* and tetraploid *G*. *hirsutum*, *G*. *barbadense* respectively. The location of *ERF* genes on D11, D01, D04, and D06 in *G. raimondii* have a good collinear relationship with the *ERF* genes present on the homologous chromosomes of *G. hirsutum* and *G. barbadense* (Fig. 4). The ERF genes on chromosome A11 in *G. arboreum* also have a good collinear relationship with the *ERF* genes present on the homologous chromosomes of *G. hirsutum* and *G. barbadense*. The synteny map revealed that small deletion, duplication and reshuffling of the chromosome may have occurred during evolution. To elucidate the divergence throughout cotton evolution, we examined the orthologous of cotton *ERF* genes between *G. arboreum, G. raimondii and G. hirsutum. In G. arboreum, a total of 113 and 117 orthologs were recognized in G. raimondii* and *G. hirsutum* respectively*.* One hundred twenty orthologs were identified between *G. raimondii,* and *G. hirsutum.* Additionally, a total of 8 and 4 paralogs for *ERF* genes were identified in *G. hirsutum* and *G. raimondii* respectively (Supplementary file 2A*).*

### Gene structure and conserved motifs of the cotton ERF gene family

**Fig. 5:**
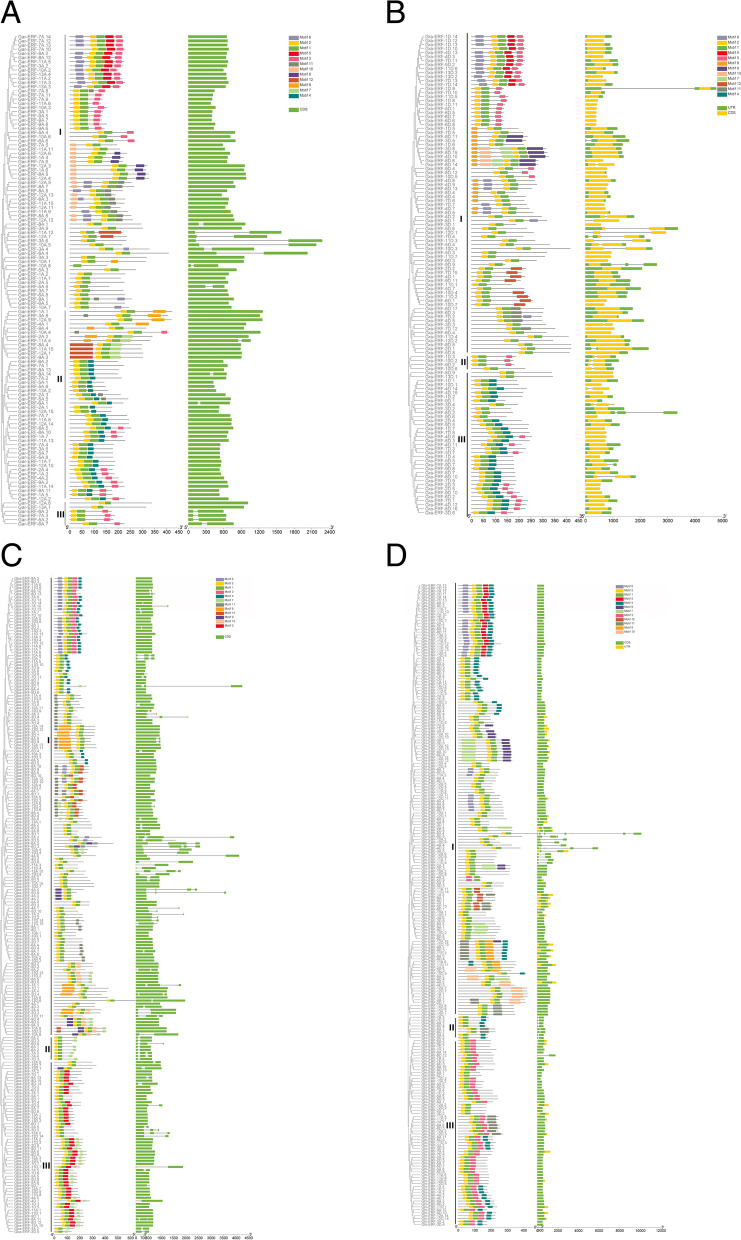
**A** Evolutionary tree with motif and gene architecture of *ERF* gene family *in G. arboreum.* **B** Evolutionary tree with motif and gene architecture of *ERF* gene family *in G. raimondii.* **C** Evolutionary tree with motif and gene architecture of *ERF* gene family *in G. barbadense.* **D** Evolutionary tree with motif and gene architecture of *ERF* gene family *in G. hirsutum*

For more understanding into the evolution and structural diversity of the *ERF* family in each cotton species, we analyzed the gene structure and conserved motif of the *ERF* genes. Motif 1 and 2 were conserved in all *ERF* genes across all four species. In *G. arboreum*, based on the evolutionary tree the *ERF* family genes are categorized into 3 groups. As shown in (Fig. 5A) only 16 genes have introns and accounting for 13.56% and the remaining 86.44% of genes are intron-less. All the *ERF* family genes have conserved exon number (1) except the 16 genes that contain introns have 2 exon numbers. The transcript length of 118 *ERF* genes of *G. arboreum, is 384–1269 bp, and the protein sequences are 127–422 aa. The* Isoelectric Point of these proteins exists between 4.292 and 10.542, and the molecular weight is between 14.323 kDa and 47.137 kDa (Supplementary file 1). To gain more insight into the divergence and functional relationship of Gar-ERF proteins, a total of 12 conserved motifs in the cotton *ERF* were recognized by MEME software, and the height of each letter in the logo was proportional to the conservation level of amino acid in all sequences analyzed. As shown in (Fig. 5A) all the Gar-ERF proteins contain motifs 1 and 2. The motifs in different groups indicated that varying degrees of divergence among them. The genes Gar-ERF-11A.12 and Gar-ERF-12A.7 were distinct in group 1 due to the presence of motif 12. The presence of motif 3 in some genes of group 2 and 3 make them different from other genes. In general, the *G. arboreum*, *ERF* proteins in the same group usually contained similar motifs, which indicates that they may play similar roles in the development and growth of *G. arboreum*.

The transcript length of 120 *ERF* genes of *G. raimondii is 399–2134 bp, and the protein sequences are 127–420 aa. The* Isoelectric Point of these proteins exists between 4.312 and 10.846, and the molecular weight is between 14.318 kDa and 47.139 kDa. 80.83% of genes were intron-less and 19.16% of genes have introns (Fig. 5B & Supplementary file 1). The gene Gra-ERF-1D.9 had the highest mean intron length 3401 bp. The 81.66, 15, and 4.16% genes have exon number 1, 2 and 3 respectively. The mean exon length ranges between 260 and 2134 bp. The motif 3, 4, 5, 6, 7, 8, 9, 10, 11 and 12 were detected in 40, 30, 13, 20, 5, 18, 7, 5, 5, and 8 genes respectively. The genes were divided into 3 groups based on the evolutionary tree. In group 1 the genes *Gra-ERF-3D.8, Gra-ERF-8D.15, Gra-ERF-4D.10, Gra-ERF-8D.6, Gra-ERF-8D.14* were distinct due to the presence of motif 10. In group 2, only one gene was unique due to the presence of motif 3 (Fig. 5B).

The transcript length of 213 *ERF* genes of *G*. *barbadense* was 339–1914 bp. The protein sequences are range between 112 and 637 aa. *The* Isoelectric Point of these proteins exists between 4.312 and 11.24, and the molecular weight is between 12.866 kDa and 71.803 kDa (Supplementary file 1). 62.44% of genes were intron-less and 37.55% of genes have introns. The gene Gba-ERF-4A.6 had the highest mean intron length 3145 bp while the gene Gba-ERF-3D.1 lowest mean intron length 27 bp. The exon number are 1–4 in all *ERF* genes of *G*. *barbadense* and the mean exon length was 119–1275 (Supplementary file 1). The motif 3, 4, 5, 6, 7, 8, 9, 10, 11 and 12 were detected in 25, 39, 45, 35, 49, 14, 13, 17, 41 and 19 genes respectively (Fig. 5C).

The transcript length of 220 *ERF* genes of *G. hirsutum*, was 327–10,114 bp. The protein sequences are range between 108 and 483 aa. *The* Isoelectric Point of these proteins exists between 4.284 and 10.846, and the molecular weight is between 11.992 kDa and 52.887 kDa. 86.36% of genes were intron-less and 13.64% of genes have introns. The gene Ghi-ERF-10A.2 had the highest mean intron length 2202 bp while the genes Ghi-ERF-5A.1 and Ghi-ERF-3D.3 have the least intron length 27 bp. Most of the genes 190, and 27 genes have 1 and 2 exon numbers (Supplementary file 1). The mean exon length is 119.5–1839. The motif 3, 4, 5, 6, 7, 8, 9, 10, 11 and 12 were detected in 60, 80, 25, 32, 16, 11, 15, 12, 26 and 8 genes respectively (Fig. 5D).

### Predicted subcellular localization

The 93.22% *ERF* genes of *G. arboreum*, were predicted in the nucleus. The genes Gar-ERF-3A.9, Gar-ERF-5A.2, Gar-ERF-6A.3 and Gar-ERF-8A.10 were found in the chloroplast. Only 3 genes namely Gar-ERF-6A.7, Gar-ERF-8A.3 and Gar-ERF-8A.6 were predicted in extracellular spaces. Only the gene was present in mitochondria (Supplementary file 1). The 92.5 and 4.16% ERF genes of *G. raimondii,* were predicted in nucleus and chloroplast respectively (Supplementary file 1). Only 1 and 3 genes were predicted in mitochondria and extracellular spaces respectively. The 89.20 and 5.63% ERF genes of *G*. *barbadense,* were predicted in nucleus and chloroplast respectively (Supplementary file 1)*.* Only 5 genes were predicted in extracellular spaces. The 91.81, 4.54, and 2.73%, *ERF* genes of *G. hirsutum*, were predicted in the nucleus, chloroplast and extracellular spaces, respectively (Supplementary file 1)*.* Only Ghi-ERF-1A.2 and Ghi-ERF-4A.4 genes were predicted in mitochondria and plasma membrane respectively.

### Analysis of putative *Cis*-acting elements in *Ghi-ERFs* promoters

**Fig. 6:**
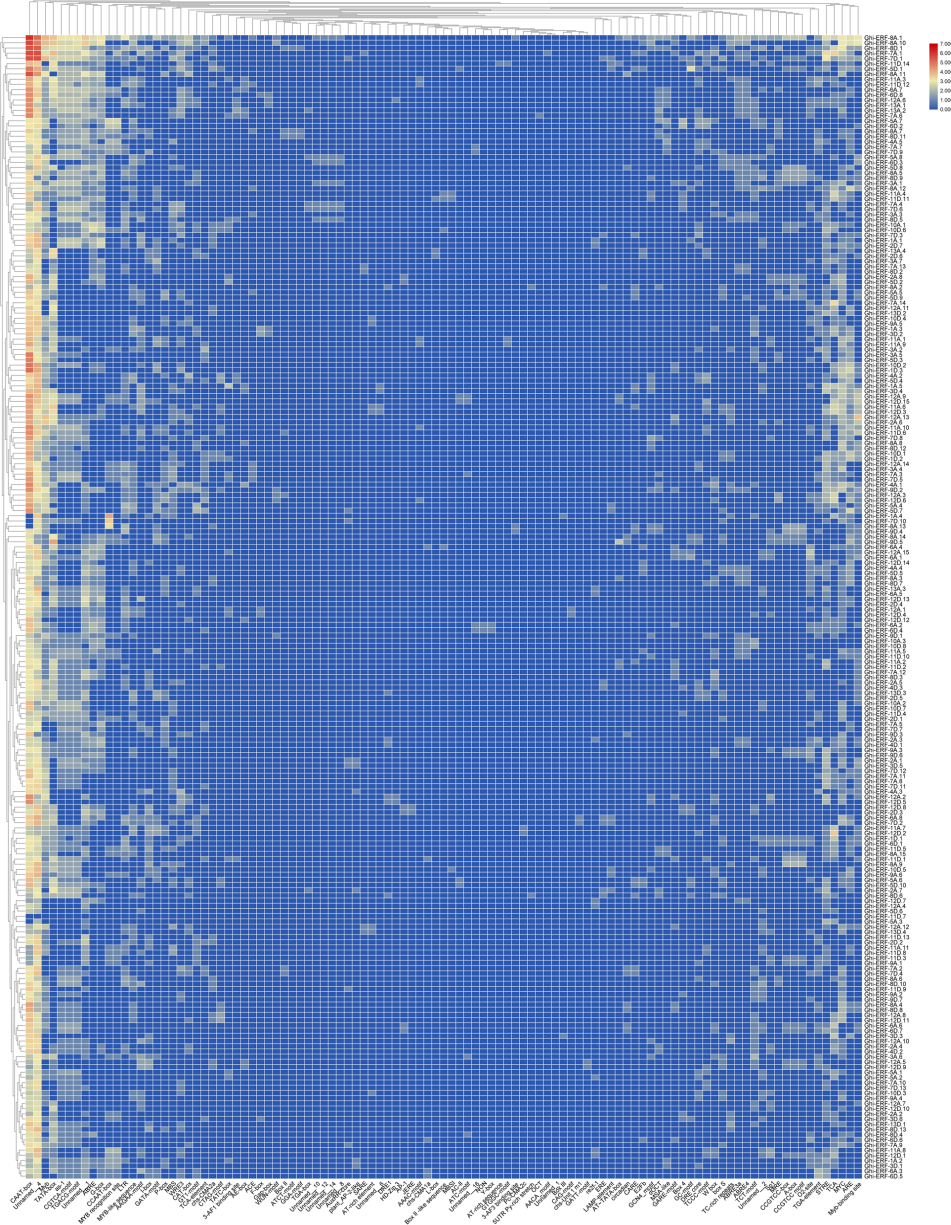
Predicted *cis*-elements in the promoter regions of *G. hirsutum ERF* genes

To investigate the further biological activity of Ghi-ERFs, the 1.5 kb upstream promoter regions of all *Ghi-ERFs* were obtained and analyzed the cis-acting regulatory elements by using the PlantCARE database. Various types of elements were found in the promoter sites of *ERF* genes in *G. hirsutum* (Fig. 6*&* Supplementary file 3). The cis-acting elements which were the binding regions of transcription factors play important role in regulating gene expression. The distribution of cis-acting elements among different genes was different, as shown in (Fig. 6*&* Supplementary file 3) a large number of cis-acting elements were predicted to be related to transcription, various hormones, and stresses response, cell cycle, and development. The promoter sites of all *Ghi-ERF* genes contained both CAAT-box and Unnamed-4 cis-acting regulatory elements. The CAAT box plays a key role in the regulation of nopaline synthase promoters [39]. The core element TATA-box was found in most of the motifs is important for transcription. There exist multiple phytohormone responsive elements including ABA response element (ABRE), ethylene-responsive element (ERE), MeJA-responsive element (CGTCA-motif, MYC and TGACG-motif), GA-responsive element (P-box), and salicylic acid-responsive element (TCA-element), suggesting that *Ghi-ERFs* expression were regulated by different phytohormones. Various light-related elements such as (Sp1, I-box, 3-AF1 binding site, TCT-motif, and Gap-box) were also observed. The stress-responsive elements were detected in the promoter of some *Ghi-ERFs*, like ARE (anaerobic induction element), STRE (stress-responsive element), LTR (low temperature-responsive elements), dehydration-responsive element (DRE1 and DRE-core), TC-rich repeats (defence and stress-responsive element), and wound-responsive element (WRE3 and WUN-motif). Some growth and development elements were also predicted, including endosperm-expression element (AACA-motif and GCN4-motif), and meristem-expression element (CAT-box and CCGTCC-motif). The results indicated that *Ghi-ERFs* are involved in plant growth and development as well as environmental stress responses.

### Expression analysis from Transcriptome data

**Fig. 7:**
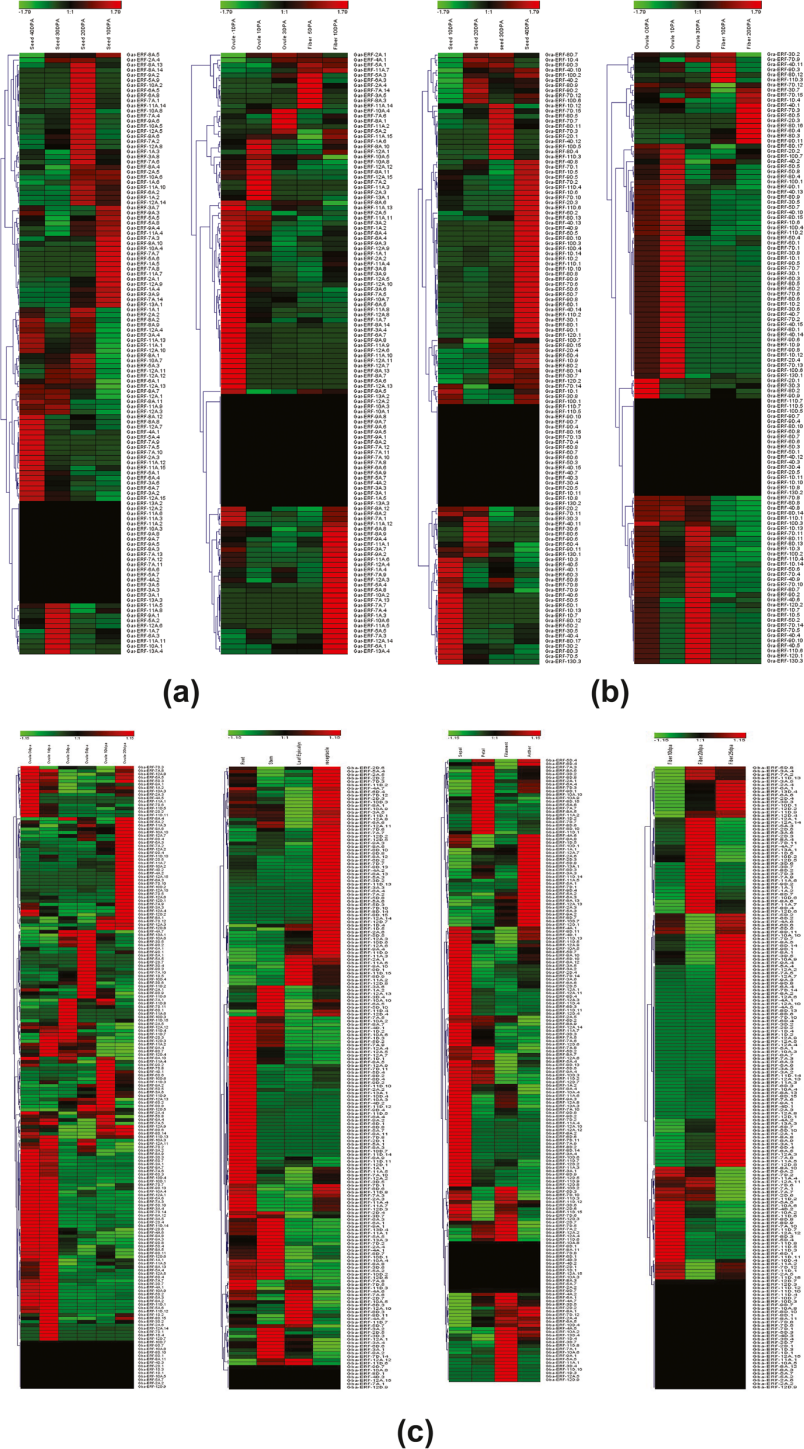
Expression patterns of *ERF* genes (**a** & **b**) in seed, ovule and fiber of *G. arboreum,* and *G. raimondii* and in (**c**) expression patterns of *ERF* genes in seed, ovule fiber, tissue and flower of *G. barbadense*

Various expression patterns of different cotton *ERF* genes were detected across different tissues like seed, stem, leaves, flower, and fiber. RNA-seq data for fiber (0 DPA (Days Post Anthesis) ovules, 1 DPA ovules, 3 DPA ovules, 5 DPA and 10 DPA fibers) and seed (10 DPA, 20 DPA, 30 DPA, and 40 DPA) of *G. arboreum,* was used for expression analysis. The Gar-ERF genes were clustered in 5 pattern groups based on the heat map of fiber and seed. Generally, a similar expression pattern was observed within groups. The genes of groups 1, 2, 3, 4 and 5 were highly expressed in ovule 5DPA, 3DPA, 1DPA, 0DPA and fiber 10DPA respectively. From group first only Gar-ERF-11A.7 were upregulated in seed, ovule and fiber development. Whereas, Gar-ERF-4A.1 and Gar-ERF-5A.1 were upregulated in fiber and ovule whilst downregulated in seed development (Fig. 7a). Twenty-one genes in *G. arboreum* did not express in any tissues.

In the case of *G. raimondii,* the genes of groups 1, 2, 4, and 5 were highly expressed in seed 20 DPA, 10 DPA, 40 DPA and 30 DPA respectively. The genes of group 3 were highly expressed in seed at 40 DPA and 30 DPA. In the case of seed, the heat map of Gra-ERF genes was clustered in six pattern groups (Fig. 7b). As for as seed and ovule were concerned, most of the genes of groups 1 and 5 were highly expressed at 20DPA whereas, 2 and 4 groups exhibited higher expression at 30 DPA, additionally, groups 3 and 6 demonstrated high expression at 40 and 10 DPA respectively. The genes of groups 1, 2, 3, 4, and 5 were highly expressed in fiber 10DPA, fiber 20DPA, ovule 1DPA, 0DPA, and 3DPA respectively. Similarly, Gar-ERF-7D.12 exhibited relatively up-regulation in seed, ovule, and fiber development (Fig. 7b). Nineteen genes have null expression across all the tissues.

The expression profile of *Gba-ERF* genes in ovule (0DPA, 1DPA, 3DPA, 5DPA, 10DPA, 20DPA) organs (root, stem, leaf epicalyx, receptacles), flower (sepal, petal, filament, anther), and fiber (10 DPA ovules, 20 DPA ovules, 25 DPA ovules) were investigated. The expression pattern of *Gba-ERF* genes in the ovule clustered the genes in 5 groups (Fig. 7c). The genes of groups 1, and 2 were expressed in ovule at 0DPA, and 20DPA respectively. The genes of group 3 were mostly expressed in 5DPA followed by 3DPA and 1DPA respectively. Most of the genes of group 4 were expressed at 10DPA. The genes of group 5 were highly expressed in 0DPA and 1DPA. The heat map of *Gba-ERF* genes in organs clustered the genes into 4 groups based on their expression pattern (Fig. 7c). The genes of 1st group were highly expressed in the receptacle and a few genes of 1st group in leaf epicalyx. Most of the genes of group 2 were highly expressed in the root. The genes of groups 3 and 4 were expressed in stem and leaf epicalyx respectively. In the case of flower and fiber the heat map also clustered the genes into 4 groups. In flower, the genes of groups 1, 2, 3 and 4 were highly expressed in petal, anther, sepal and filament respectively (Fig. 7c). Most of the genes of groups 1 and 3 were highly expressed in fiber at 25DPA but the genes Gba-ERF-5D.8, Gba-ERF-3A.4, Gba-ERF-7A.2, Gba-ERF-11D.13 from 1st group were expressed in fiber at 20 DPA. The genes of group 2 were expressed in fiber at 20 DPA. The genes of group 4 were highly expressed in fiber at 10 DPA (Fig. 7C). Specifically, Gba-ERF-2A.4 exhibited up-regulation in ovule development, fiber development, root, stem, petal, filament, and anther.

**Fig. 8:**
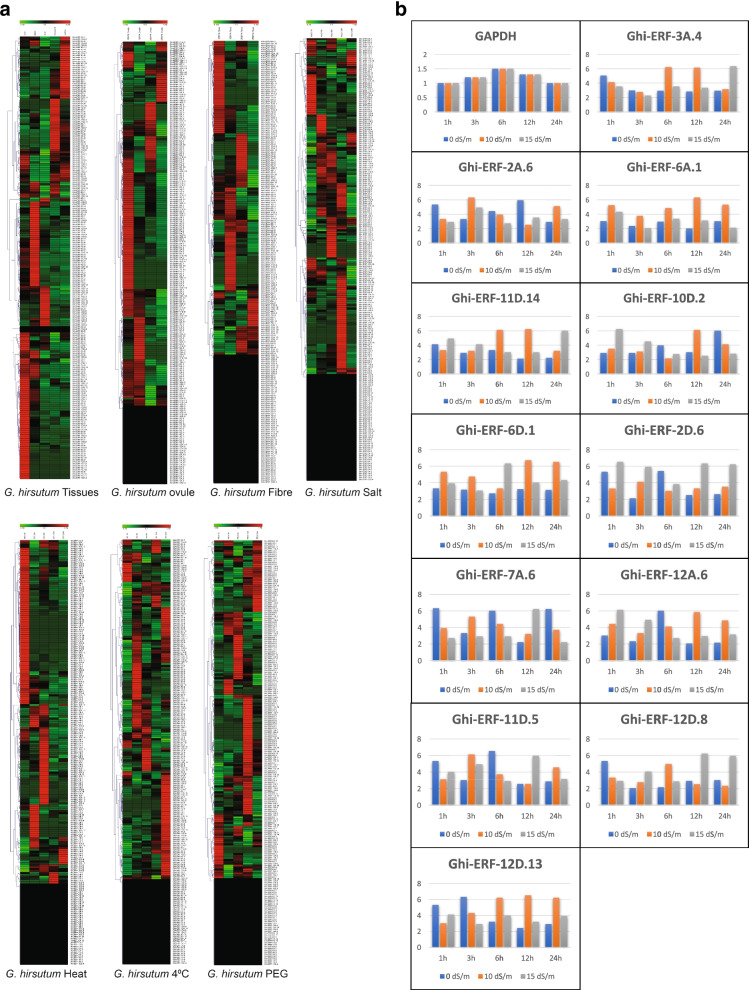
**A** Expression patterns of *ERF* genes in tissue, ovule, fiber and (salinity, heat, cold and PEG) conditions at for (1 h, 3 h, 6 h, 12 h and 24 h) in *G. hirsutum.* **B** Relative expression patterns of twelve *Ghi-ERF* genes under NaCl treatment analyzed by qRT-PCR

In *G. hirsutum*, the *Ghi-ERF* genes expression was examined in organs (root, stem, leaf, filaments, anther), ovule (10DPA, 15DPA, 20DPA, 25DPA), fiber (10DPA, 15DPA, 20DPA, 25DPA), salinity, heat, cold, and PEG stress for (1 h, 3 h, 6 h, 12 h and 24 h) respectively. Based on *Ghi-ERF* genes expression in organs (root, stem, leaf, filaments and anther), the genes were clustered in 5 groups (Fig. 8A). Most of the genes of 1, 2, 3, 4 and 5 were highly expressed in anther, filaments, stem, leaf and root respectively. An ovule and fiber, based on heat map Ghi-ERF genes were clustered in 4 expression pattern groups (Fig. 8A). For ovule, most of the genes of groups 1, 2, 3 and 4 were highly expressed at 25DPA, 20DPA, 10DPA, and 15DPA respectively. Most of the genes of groups 1, 2, 3 and 4 were highly expressed in fiber at 10DPA, 20DPA, 15DPA, and 25DPA respectively. Under salt stress, the heat map clustered the genes in 3 groups (Fig. 8A). Most of the Ghi-ERF genes of group 1 were highly expressed at NaCl 1 h, while some genes of group 1 were also expressed at NaCl 24 h. The Ghi-ERF genes of 2nd group were differentially expressed at NaCl 3 h, NaCl 6 h, and NaCl 12 h. Most of the genes of group 3 were highly expressed at NaCl 12 h. The heat map of Ghi-ERF genes in heat stress divides the genes into three groups. The genes of group 1 were highly expressed at 37 C 1 h. Most of the genes of 2nd and 3rd groups were highly expressed at 37 C 6 h and 37 C 3 h respectively (Fig. 8A). Some genes of group 3 were also expressed at 37 C in 24 h and 12 h. The expression of *ERF* genes under cold and PEG stress the heat map clustered these genes in 5 groups. In the case of cold stress, most of the genes of group 1 were expressed at 4C in 1 h and 12 h. The genes of groups 2, 3, 4 and 5 were highly expressed at 4C in 24 h, 3 h, 6 h and 24 h respectively. In the case of PEG stress, most of the genes of groups 1, 2, 3, 4 and 5 were highly expressed in 24 h, 6 h, 3 h, 12 h and 1 h respectively (Fig. 8A). Interestingly, Ghi-ERF-2D.6, Ghi-ERF-6D.1, Ghi-ERF-11D.5, Ghi-ERF-12D.13, and Ghi-ERF-7A.6 demonstrated comparatively up-regulation under various tissues.

### Examination of the expression of ERF genes by qPCR

The quantitative measurement of mRNA expression in the leaf of cultivar Eagle-2 at different salt stress levels after regular intervals was performed. GAPDH was used as an internal control of gene normalization. In Fig. 8B, the genes Ghi-ERF-2D.6, Ghi-ERF-12D.13, Ghi-ERF-6D.1, Ghi-ERF-7A.6 and Ghi-ERF-11D.5 showed relatively higher expression at 15 dS/m, 10dS/m, 0dS/m, 10 dS/m and 10 dS/m after regular intervals of 1, 3, 6, 12 and 24 h respectively. This expression level of the genes is the highest among all the genes and these five genes showed significant tolerance against salt stress. Primers are listed in Supplementary file 4.

## Discussion

In plants, the (AP2/EREBP) superfamily is one of the important and largest transcription factor (TF) families. AP2/ERF domain specifies different members of the (AP2/EREBP) family. The domain consists of up to 60–70 amino acids [4]. These members have necessary roles in the growth and development of plants, and in responding to a variety of environmental stresses involving heat, drought, salinity, cold and various pathogen infections. The roles are exerted either by direct response to the stresses or by regulation of expression of target genes downstream [40]. Numerous studies have recognized different numbers of ERF family members in plants; like in soybean 120 ERF family members [41], cucumber 103 (*Hu & Liu, 2011*) tomato 85 [42], rice 139 [43] and 147 Arabidopsis [44] respectively.

Arabidopsis, used as the model plant comprises 147 *AP2/EREBP* genes divided into 4 subfamilies which are; *APETALA2 (AP2)*, Ethylene-Responsive Factor *(ERF)*, Dehydration-Responsive Element Binding protein (*DREB*), and Related to ABI3/VP1 (*RAV*) subfamilies [45]. Subfamily members of ERF and DERB containing single conserved domain AP2 and making the largest groups in superfamily *AP2/EREBP*, perform important roles in various plant processes and variety of stress responses. The sequences containing one AP2 domain have the biggest number of members in the superfamily *AP2/EREBP*, which have been further divided into 2 major subfamilies; *ERF* subfamily and *DREB* subfamily [46].

Cotton is an important economic crop in the world providing the leading share of the world’s natural textile fiber and a substantial amount of edible oil. Studies revealed the formation of the allotetraploid *Gossypium* species occurred about 1–1.5 million years back through a polyploidization event that involved two species; one being A-genome species called *G. arboreum* and the other was paternal D-genome species named *G. raimondii* [18].

WGD or polyploidy is being acknowledged as a source of evolution in plants, a significant force resulting in enormous silencing and removal of duplicated genes [47]. In this study, we identified 118, 120, 213, 220 ERF genes in *G. arboreum, G. raimondii, G. barbadense* and *G. hirsutum* respectively. The evolutionary tree showed that more ERF proteins appeared in pairs and clustered together with 2 Gba-ERF, 2 Ghi-ERF, 1 Gar-ERF and 1 Gra-ERF which supported the cotton species polyploidization event that occurred 1.5 million years ago [48].

The tetraploid cotton should contain ERF genes in a number equal to *ERF* genes contained in *G. arboreum* plus ERF genes in *G. raimondii.* Though the number of *ERF genes actually* in allotetraploids (*G. barbadense* 213 and *G. hirsutum* 220) appeared lesser than those in two diploids (*G. arboreum* 118 and *G. raimondii* 120), implying that the chromosomes doubling and then genome rapid sequence arrangement would produce gene loss of different degrees in polyploidization process [18]. Gene duplication is a significant source of evolution in the genome and turn genetic system by producing new gene subfamilies [49]. Polyploidy, segmental, and Tandem duplications chiefly help to generate new gene families [49]. ERF genes in *G. arboreum,* (13), *G. barbadense* (15), *G. hirsutum* (7) and *G. raimondii,* (13) have tandem duplications. Segmental duplications frequently take place in plants as most plants occur as diploidized polyploids [50]. In *G. arboreum* (92), *G. hirsutum* (208), *G. barbadense* (182), and *G. raimondii* (74) ERF genes have undergone segmental or WGD duplications.

There’s a possibility of deletion of some of the pre-existing genes or newly generated genes during the process of cotton evolution. Collinearity and chromosomal locations showed the significant role of segmental duplications in ERF genes expansion in cotton which was following the results reported in rice, *B. napus*, *A. thaliana* and other plants [10, 42, 51, 52]. The synteny analysis results could be used to reveal the evolutional and functional connections among the cotton species. In these studies, 237, 105 duplication gene pairs were established between diploid *G*. *arboreum* and tetraploid *G*. *barbadense, G*. *hirsutum* correspondingly. And there were 393, 307 duplication gene-pairs established between diploid *G*. *raimondii* and tetraploid *G*. *hirsutum*, *G*. *barbadense* correspondingly. Our study analysis showed a higher homology between ERF genes of tetraploid and diploid cotton species. Some *ERF* genes did not find any orthologous gene pairs, which could be attributed to chromosomes rearrangement or fusion during the evolution [53, 54].

Gene structure analysis plays a crucial role in revealing the function of genes [51]. Here, our results suggested that 13.56, 19.16, 37.55 and 13.64% genes possess introns in *G. arboreum, G. raimondii, G. barbadense* and *G. hirsutum* respectively. The ERF members demonstrated similar gene structures within the same group. Loss of introns occurred more rapidly in genes than intron acquisition after segmental duplication [55]. Also, some studies revealed that intron number and distribution were related to evolution in plants [56] in a way that introns possibly have been lost during evolution from ERF family genes in higher plants. Our results showed that 86.44, 80.83, 62.44, and 86.36% ERF genes have no introns which are similar to the status in *Arabidopsis*, cucumber, and rice [57–59]. Transcriptional output can be delayed due to long multiple introns, possibly causing suppression of genes expression in adverse conditions. Contrarily, the genes containing small or fewer introns might have efficient expression while responding to stress environments [60]. Therefore many intron-less *ERF* genes might react quickly to the external environment variations [51]. The transcription factors domains and motifs perform essential roles during transcriptional activity, proteins interaction, and DNA binding [61].

Here, a total of 12 conserved motifs in the *G. arboreum, G. raimondii, G. barbadense* and *G. hirsutum* for ERF genes were identified. Gene function diversity could be affected by different numbers and types of motifs present in ERF proteins, in all four species. Motif 1 and motif 2 in all ERF genes of cotton were highly conserved. The ERF family members, in general, shared similar structures of gene and motif compositions within the same group, which proposes the possibility of their similar roles in plant growth and development. In promoter sites, *Cis*-regulatory elements of genes play key roles in plant stress responses [62]. Various *cis*-acting elements responsible for stresses, phytohormones, growth and development were discovered in the promoter sites of *Ghi-ERFs*, stipulating the potential role of *Ghi-ERFs* in regulating several responses to phytohormones, environmental stresses, and development. For example, (ABRE, CGTCA-motif, MYC, TGACG-motif, ERE, P-box, and TCA-element), (ARE, STRE, LTR, DRE1, DRE-core, TC-rich repeats, WRE3 and WUN-motif) and (CAT-box, CCGTCC-motif, AACA-motif and GCN4-motif), are associated to phytohormones, stresses, growth and development respectively [63–67]. Based on these results, we speculate that the *Ghi-ERFs* genes are probably involved in abiotic stress responses.

The study of expression patterns of genes is used to predict the function of genes [53]. In this study the ERF genes showed high expression in ovule (0DPA, 1DPA, 3DPA, 5DPA, 10DPA, 20DPA) organs (root, stem, leaf epicalyx, receptacles), flower (sepal, petal, filament, anther) and fiber (10 DPA, 20 DPA, 25 DPA) across the species, suggesting that these genes have key roles in growth and development of cotton plant. Moreover, the expression of cotton ERF in different tissues or diverse stages, showed that these genes could be more stable than those that only expressed in specific tissues or one stage of an organ. Most of the ERF genes in *G. hirsutum* were also expressed in salt and heat stress.

*Ghi-ERF-2D.6, Ghi-ERF-12D.13, Ghi-ERF-6D.1, Ghi-ERF-7A.6 and Ghi-ERF-11D.5* identified as candidate genes against salinity tolerance in upland cotton by RT-PCR. According to previous studies, some *ERF* genes were involved in various stresses responses in plants, such as high-salt, low-temperature, and drought stress [46, 57]. Overall, the above findings provide a foundation to further investigate the potential function of cotton *ERF* genes. These analyses are not only helpful in selecting valuable candidate *ERF* genes for further functional studies but also has important implication for genetic improvement for agricultural production and stress tolerance in cotton crop.

## Conclusions

In this study, a genome-wide analysis of cotton *ERF* genes was performed. The 671 *ERF* genes in four species were identified and classified in detail. Protein lengths, molecular weights, and theoretical isoelectric points of cotton *ERF* vary greatly. The evolutionary characteristics, expression patterns of *ERF* genes in various cotton organs and growth stages, and their response to abiotic stress were studied. The expression profile analysis suggested that the *ERF* gene family of upland cotton may be important in stress responses. We proved that *Ghi-ERF-2D.6, Ghi-ERF-12D.13, Ghi-ERF-6D.1, Ghi-ERF-7A.6* and *Ghi-ERF-11D.5* are responsive to salt stress by qRT-PCR. These results will be helpful to understand the biological role of the *ERF* genes in cotton growth and development.

## Supplementary Information

**Additional file 1.**

**Additional file 2.**

**Additional file 3.**

**Additional file 4.**

**Additional file 5.**

## Data Availability

We did not generate any sequencing data during this experiment. We have used already published RNA-seq. Data with FPKM values from NCBI database to select our candidate genes to perform RT_PCR. And their bio-project numbers are already mentioned in the manuscript. The detailed links are given below. We have used all the genomes and their transcriptomic data from Cotton Functional Genomics Database (CottonFGD) (https://cottonfgd.org). Moreover, the raw expression reads data from various anatomical tissues and various time-points of ovule and fiber of *G. arboreum* cultivar Shixiya1 and *G. hirsutum* accession TM-1 were retrieved from NCBI-Bioproject PRJNA594268. Furthermore, RNA-seq expression data of *G. hirsutum* accession TM-1 and cultivar Hai7124 were retrieved from NCBI-Bioproject PRJNA490626 for *G. barbadense*. Similarly, raw expression data of strain Ulbr of *G. raimondii* were obtained from NCBI-Bioproject PRJNA171262.
