# Supplementary material for: Genome-wide characterization and expression analysis of Erf gene family in cotton
Source: BMC Plant Biol. 2022 Mar 22;22:134. doi: 10.1186/s12870-022-03521-z (PMC8939120; doi:10.1186/s12870-022-03521-z)
Supplement: Supplementary file 4 — Additional file 4. [file 12870_2022_3521_MOESM4_ESM.docx]

**Supplementary File 4: List of primers used for qRT-PCR.**

| **Sr. #** | **GeneID** | **Fprimer** | **Rprimer** |
| --- | --- | --- | --- |
| 1 | Ghi-ERF-6A.1 | CTTACTCCACACCCATAGCCGC | GTCAGGGAAGTTGAGCCTAGCG |
| 2 | Ghi-ERF-2A.6 | TCTTCTTCTCCCTCACAGCCCA | GGGGTGAGTTCGTTAAGCCCAA |
| 3 | Ghi-ERF-3A.4 | GAGGAGAAGGGAAGTGTGACGC | ACATAACCGCAAGCCCGGAATA |
| 4 | Ghi-ERF-6A.1 | GGCCATGGAAGGTGACTGTTGT | AGAACACGGCGGTATCGTAAGC |
| 5 | Ghi-ERF-7A.6 | CCTCCTCCAACACCCCAGTTTC | TCGTGGCGGTGGAGGTTATTTC |
| 6 | Ghi-ERF-12A.6 | CGGCGTTTTGTTTGAGAGGACG | AGCAGTGGACGTCAACGGTATG |
| 7 | Ghi-ERF-2D.6 | AGCGTGGAGTCAACACCTTGTC | CATTAGCGACTCCACCACCCAG |
| 8 | Ghi-ERF-2A.6 | GCTCCCCATTGAACAGTAGTACCC | AACGGGTGAGTTGGTTAAGCCC |
| 9 | Ghi-ERF-6D.1 | GGCCATGGAAGGTGACTGTTGT | ATTTCAGCCACCCATTTCCCCC |
| 10 | Ghi-ERF-7A.6 | CTTTCGAAACCGCCATCGAAGC | CCCGTTGTCGTTACGTTTTCCG |
| 11 | Ghi-ERF-10D.2 | AATCACAGCCCTAGCCGGAGTA | GATCCGTACCCGATGACTGCAC |
| 12 | Ghi-ERF-11D.5 | TCTGACCCGAATTGCCGAATCC | CCTTGGCAGCATCGACATCAGT |
| 13 | Ghi-ERF-11D.14 | TGGCTAGGCACTTTCGATTCGG | CGTCGCCGAAAGCTGGGATATT |
| 14 | Ghi-ERF-12D.8 | CTTCACCATCCTCGGTGCATGA | CAGCTCTTGTCCAACTGAGCCA |
| 15 | Ghi-ERF-12D.13 | GAGGAAACGGAGCAGAGACGAC | CCCCTTCGTGTCGTTATCGCAA |
